# Supplementary material for: Discovery of a New Xanthone against Glioma: Synthesis and Development of (Pro)liposome Formulations
Source: Molecules. 2019 Jan 23;24(3):409. doi: 10.3390/molecules24030409 (PMC6384625; doi:10.3390/molecules24030409)
Supplement: Supplementary file 1 [file molecules-24-00409-s001.pdf]

Supplementary material

Discovery of a New Xanthone Against Glioma: Synthesis and Development of (Pro)liposome Formulations

Ana Alves [1,2,†], Marta Correia-da-Silva[2,3,†], Claúdia Nunes[4], João Campos[1], Emília Sousa[2,3*], Patrícia M.A. Silva[5], Hassan Bousbaa[3,5], Francisca Rodrigues[6], Domingos Ferreira[1], Paulo C. Costa[1*], Madalena Pinto[2,3]

[1] UCIBIO, REQUIMTE, Laboratory of Pharmaceutical Technology, Faculty of Pharmacy, University of Porto, Rua de Jorge Viterbo Ferreira, 228, 4050-313 Porto, Portugal; anadaniela92@hotmail.com (A.A.); jcampos@ff.up.pt (J.C.); domingos@ff.up.pt (D.F.); pccosta@ff.up.pt (P.C.C.);

[2] Laboratory of Organic and Pharmaceutical Chemistry, Department of Chemical Sciences, Faculty of Pharmacy, University of Porto, Rua Jorge Viterbo Ferreira, 228, 4050-313 Porto, Portugal; m_correiadasilva@ff.up.pt (M.C.S.); esousa@ff.up.pt (E.S.); madalena@ff.up.pt (M.P.)

[3] Interdisciplinary Centre of Marine and Environmental Research (CIIMAR), University of Porto, Terminal de Cruzeiros do Porto de Leixões Avenida General Norton de Matos P 4450-208 Matosinhos, Portugal;

[4] LAQV, REQUIMTE, Departamento de Ciências Químicas, Faculdade de Farmácia, Universidade do Porto, Rua de Jorge Viterbo Ferreira, 228, 4050-313 Porto, Portugall; cdnunes@ff.up.pt (C.N.);

[5] CESPU, Institute of Research and Advanced Training in Health Sciences and Technologies (IINFACTS), Rua Central de Gandra, 1317, 4585-116 Gandra, Portugal; patricia.silva@cespu.pt (P.M.A.S); hassan.bousbaa@iucs.cespu.pt (H.B.);

[6] REQUIMTE/LAQV, Instituto Superior de Engenharia do Porto, Instituto Politécnico do Porto, Portugal franciscapintolisboa@gmail.com (F.R.)

* Correspondence: pccosta@ff.up.pt (P.C.C), Tel.: +351220428620; esousa@ff.up.pt (E.S.), Tel.: +351220428689.

† These authors contributed equally to this work.

Figure S1.

Compound **2**

^1H NMR

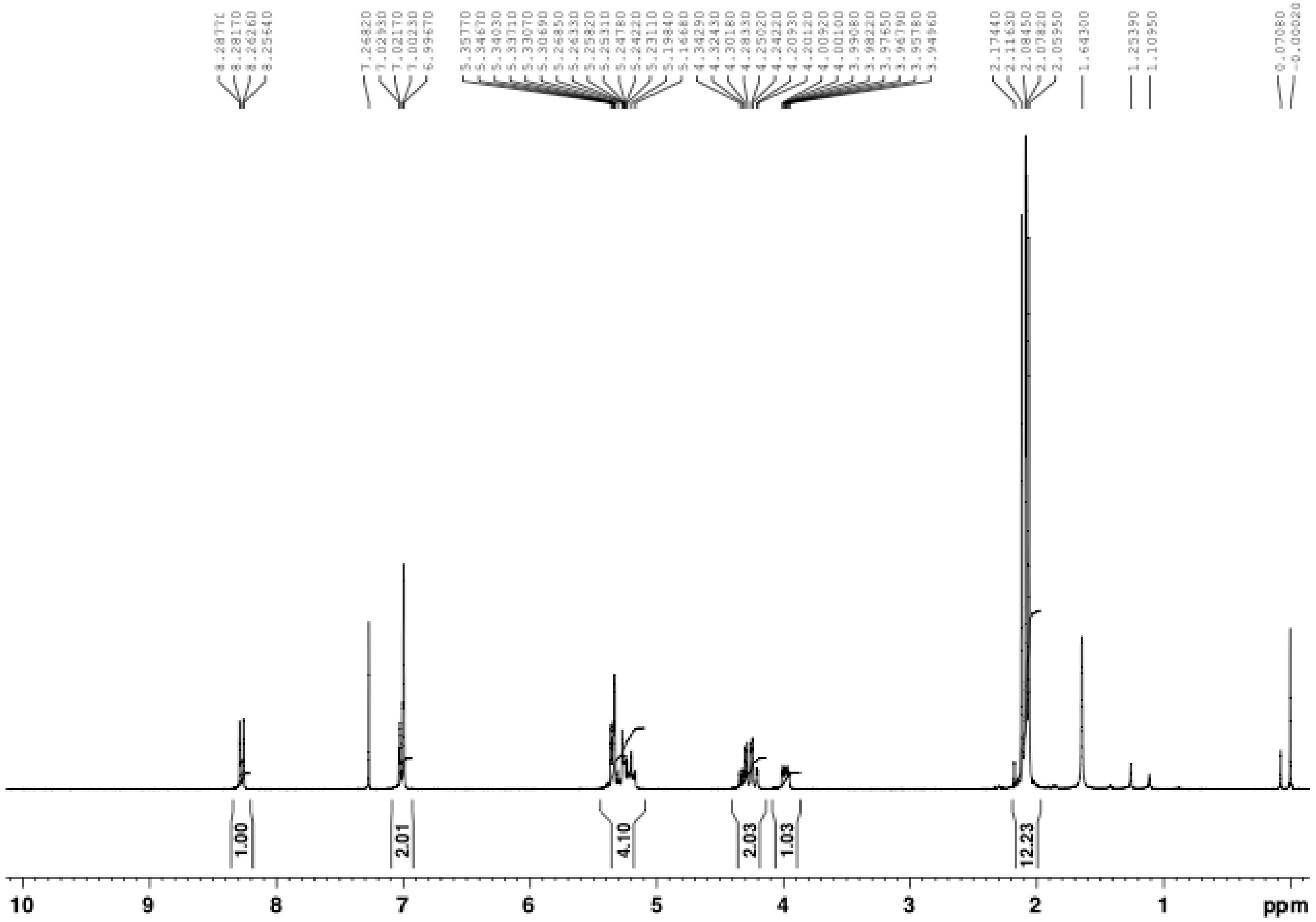

^{13}C NMR

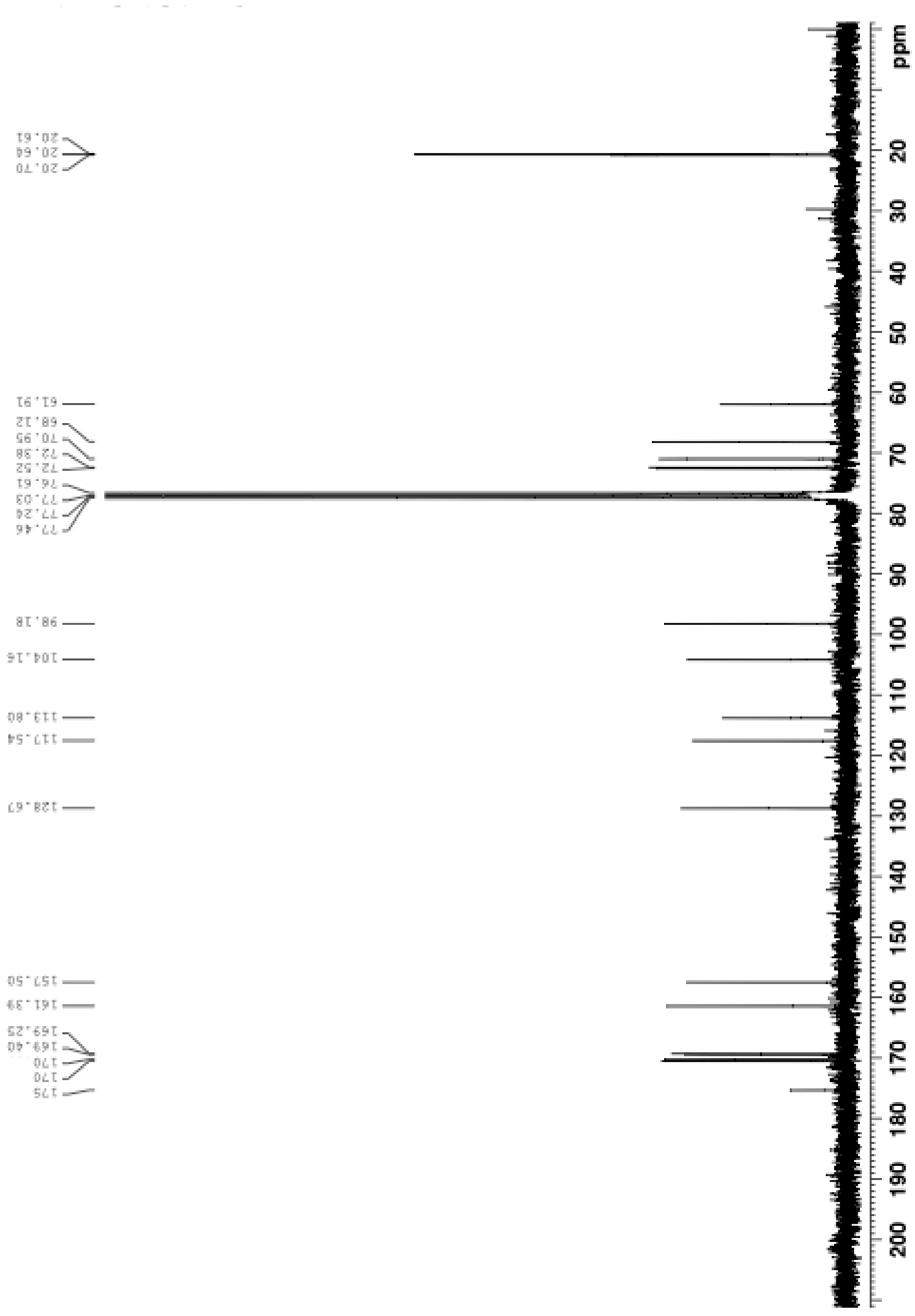

Compound **2**

Figure S2.

Mass Spectrum List Report

Analysis Info **Electrospray (ESI)**
Analysis Name JRMP1401382_000001.d
Instrument apex Qe

Acquisition Parameter
Capillary Exit 300.0 V
Skimmer 1 20.0 V

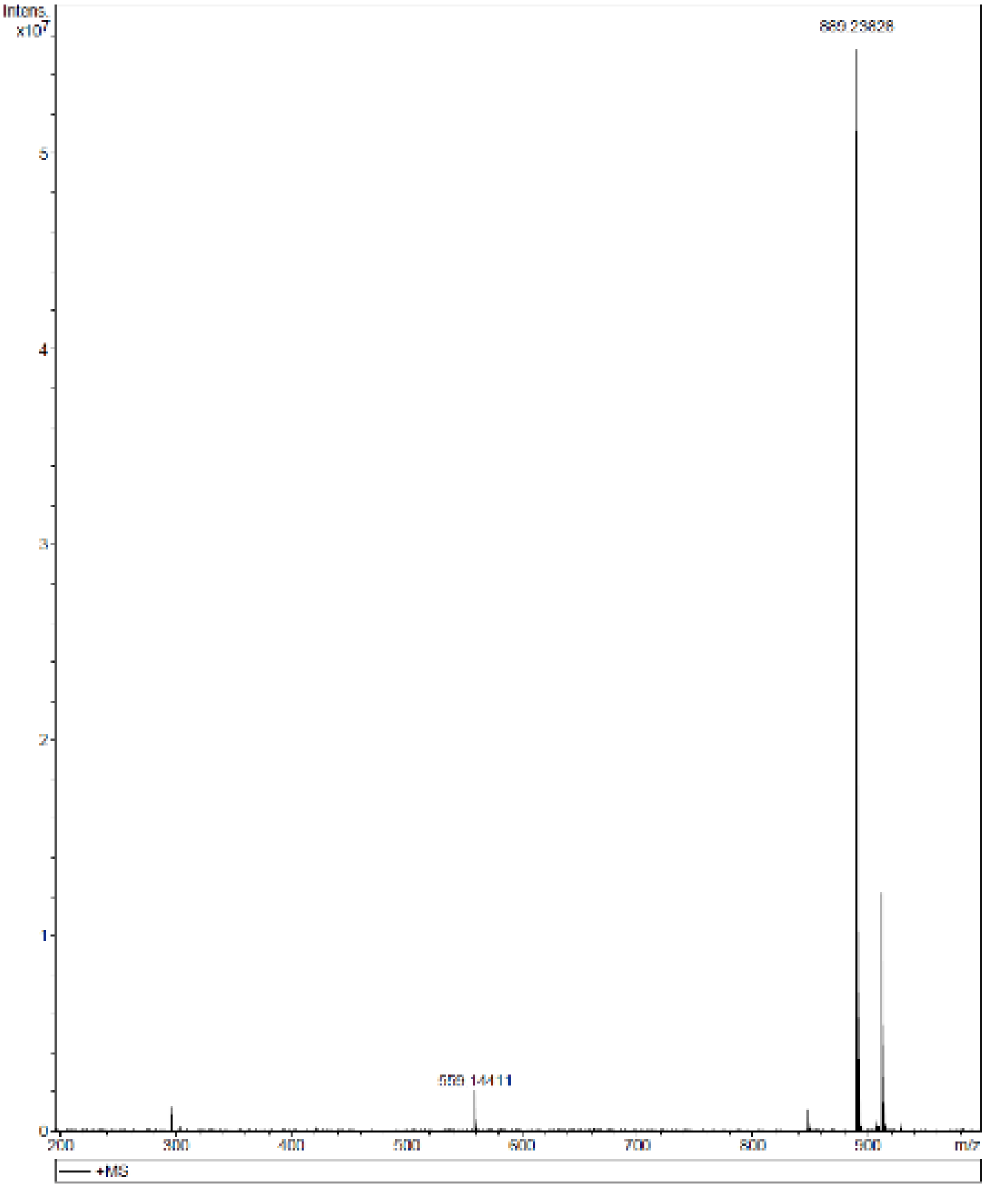

Compound **2**

Mass Spectrum Molecular Formula Report

Analysis Info **Electrospray (ESI)**

Analysis Name D:\Data\AEMPRESAS\JRMP1401382_000001.d

Instrument apex Qe

Acquisition Parameter

Capillary Exit 300.0 V

Skimmer 1 20.0 V

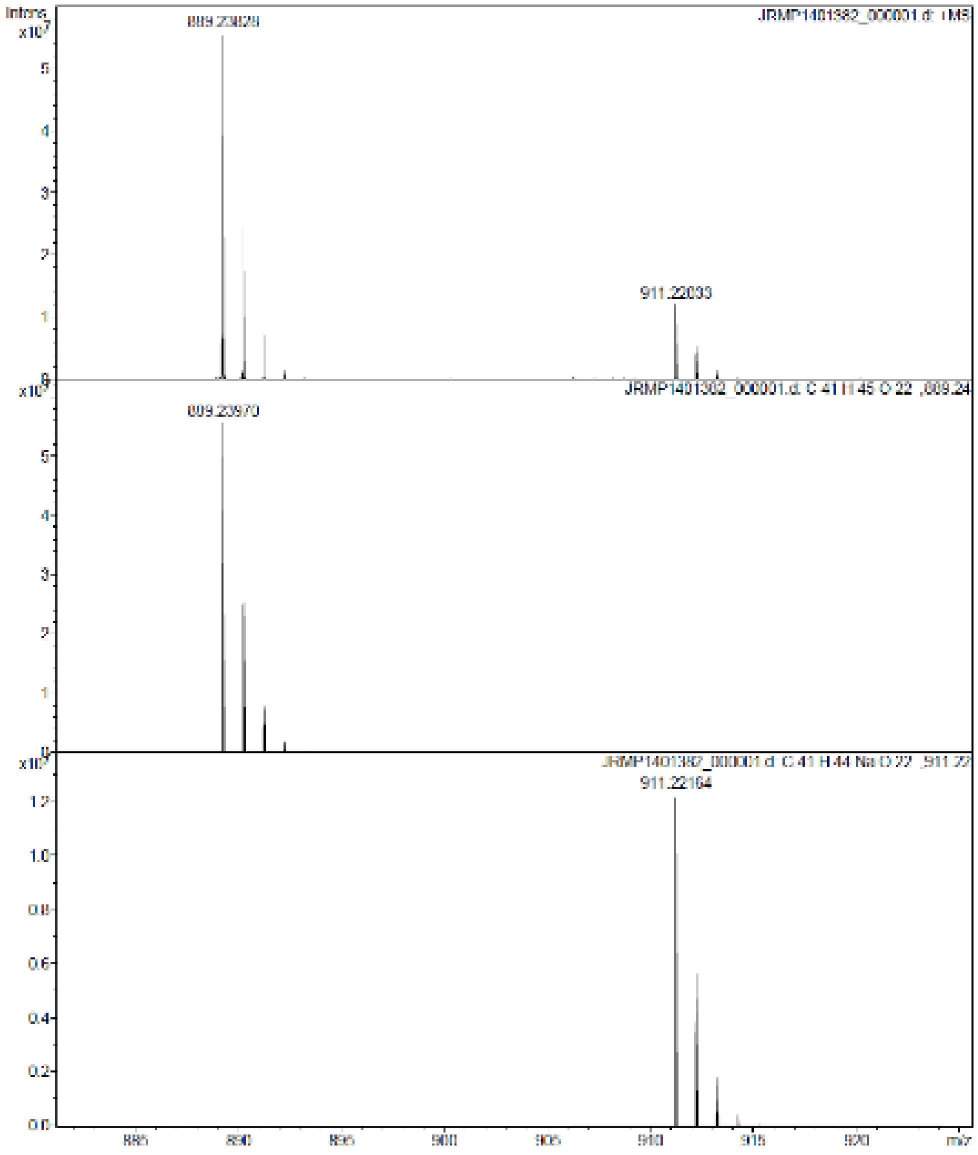

Compound 2

Mass Spectrum Molecular Formula Report

Meas. m/z	#	Formula	Score	m/z	err [mDa]	err [ppm]	mSigma	rdb	e⁻ Conf	N-Rule
889.23828	1	C 41 H 45 O 22	100.00	889.23970	1.41	1.59	20.0	19.5	even	ok
911.22033	1	C 41 H 44 Na O 22	100.00	911.22164	1.31	1.44	68.0	19.5	even	ok
	2	C 43 H 43 O 22	9.83	911.22405	3.71	4.08	74.1	22.5	even	ok
	3	C 50 H 39 O 17	15.86	911.21818	-2.16	-2.37	97.8	31.5	even	ok

Table S1.

Multiple Comparisons for Table 2 (Mean diameter)

Dependent Variable: Mean Diameter

Tukey HSD

(I) Type	(J) tipo	Mean Difference (I-J)	Std. Error	Sig.	95% Confidence Interval Lower Bound	Upper Bound
Water - manual	Water - sonication	36.03333*	8.54618	.012	8.6655	63.4012
	PBS - manual	-44.46667*	8.54618	.004	-71.8345	-17.0988
	PBS - sonication	25.53333	8.54618	.068	-1.8345	52.9012
Water - sonication	Water - manual	-36.03333*	8.54618	.012	-63.4012	-8.6655
	PBS - manual	-80.50000*	8.54618	.000	-107.8679	-53.1321
	PBS - sonication	-10.50000	8.54618	.628	-37.8679	16.8679
PBS - manual	Water - manual	44.46667*	8.54618	.004	17.0988	71.8345
	Water - sonication	80.50000*	8.54618	.000	53.1321	107.8679
	PBS - sonication	70.00000*	8.54618	.000	42.6321	97.3679
PBS - sonication	Water - manual	-25.53333	8.54618	.068	-52.9012	1.8345
	Water - sonication	10.50000	8.54618	.628	-16.8679	37.8679
	PBS - manual	-70.00000*	8.54618	.000	-97.3679	-42.6321

*. The mean difference is significant at the 0.05 level.

Table S2.

Multiple Comparisons for Table 3 (Mean diameter)

Dependent Variable: Mean Diameter

Tukey HSD

(I) Type	(J) Type	Mean Difference (I-J)	Std. Error	Sig.	95% Confidence Interval Lower Bound	95% Confidence Interval Upper Bound
Prolipo	Prolipo + XGAC	-20.7333	20.37740	.745	-85.9889	44.5223
	Lipo	61.5333	20.37740	.065	-3.7223	126.7889
	Lipo + XGAC	76.2667*	20.37740	.024	11.0111	141.5223
Prolipo + XGAC	Prolipo	20.7333	20.37740	.745	-44.5223	85.9889
	Lipo	82.2667*	20.37740	.016	17.0111	147.5223
	Lipo + XGAC	97.0000*	20.37740	.006	31.7444	162.2556
Lipo	Prolipo	-61.5333	20.37740	.065	-126.7889	3.7223
	Prolipo + XGAC	-82.2667*	20.37740	.016	-147.5223	-17.0111
	Lipo + XGAC	14.7333	20.37740	.885	-50.5223	79.9889
Lipo + XGAC	Prolipo	-76.2667*	20.37740	.024	-141.5223	-11.0111
	Prolipo + XGAC	-97.0000*	20.37740	.006	-162.2556	-31.7444
	Lipo	-14.7333	20.37740	.885	-79.9889	50.5223

Based on observed means.

The error term is Mean Square(Error) = 622.857.

*. The mean difference is significant at the .05 level.

Figure S3.

Simple Bar of Entrapment Efficiency (EE) vs Type of particle (proliposome or liposome with XGAC)

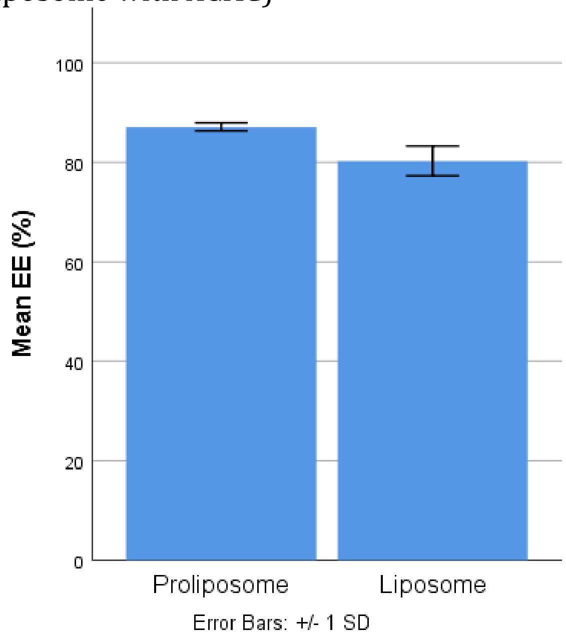

Table S3.

Levene test and t-test for Entrapment Efficiency (EE) (Independent Samples)

		Levene's Test for Equality of Variances		t-test for Equality of Means						
									95% CI of the Difference	
		F	Sig.	t	df	Sig. (2-tailed)	Mean Difference	Std. Error Difference	Lower	Upper
EE	Equal variances assumed	8.0582	.0469	3.8661	4.000	.0181	6.8900	1.7821	1.9420	11.8380
	Equal variances not assumed			3.8661	2.263	.0498	6.8900	1.7821	.0151	13.7649

Table S4.

Multiple Comparisons for Figure 5 (XGAC)

Dunnett t (2-sided)[a]

Dependent Variable	(I) Conc	(J) Conc	Mean Difference (I-J)	Std. Error	Sig.	95% Confidence Interval	
						Lower Bound	Upper Bound
U251	.10	.00	-.38862	5.24743	1.000	-15.5562	14.7790
	1.00	.00	-16.65584*	5.24743	.031	-31.8234	-1.4883
	10.00	.00	-44.90061*	5.24743	.000	-60.0682	-29.7330
	100.00	.00	-75.77833*	5.24743	.000	-90.9459	-60.6107
U373	.10	.00	-4.47331	6.01732	.868	-21.8662	12.9196
	1.00	.00	-12.68692	6.01732	.174	-30.0799	4.7060
	10.00	.00	-26.90202*	6.01732	.004	-44.2950	-9.5091
	100.00	.00	-69.16755*	6.01732	.000	-86.5605	-51.7746
U87MG	.10	.00	-3.72778	3.64110	.704	-14.2523	6.7967
	1.00	.00	-10.78675*	3.64110	.044	-21.3113	-.2622
	10.00	.00	-16.57165*	3.64110	.004	-27.0962	-6.0471
	100.00	.00	-25.18760*	3.64110	.000	-35.7121	-14.6631

*. The mean difference is significant at the 0.05 level.

a. Dunnett t-tests treat one group as a control, and compare all other groups against it.

Multiple Comparisons for Figure 5 (Lipo)

Dunnett t (2-sided)[a]

Dependent Variable	(I) Conc	(J) Conc	Mean Difference (I-J)	Std. Error	Sig.	95% Confidence Interval	
						Lower Bound	Upper Bound
U251	.10	.00	-1.07184	1.39371	.854	-5.1003	2.9567
	1.00	.00	-.69795	1.39371	.961	-4.7265	3.3305
	10.00	.00	-2.41743	1.39371	.302	-6.4459	1.6111
	100.00	.00	-7.76538*	1.39371	.001	-11.7939	-3.7369
U373	.10	.00	-1.96644	1.84173	.676	-7.2899	3.3570
	1.00	.00	-4.14116	1.84173	.140	-9.4646	1.1823
	10.00	.00	-4.46241	1.84173	.107	-9.7859	.8611
	100.00	.00	-6.02775*	1.84173	.027	-11.3512	-.7043
U87MG	.10	.00	-.33334	1.55038	.998	-4.8147	4.1480
	1.00	.00	-.98489	1.55038	.917	-5.4662	3.4964
	10.00	.00	-.60458	1.55038	.984	-5.0859	3.8768
	100.00	.00	-.22621	1.55038	1.000	-4.7075	4.2551

Multiple Comparisons for Figure 5 (Lipo + XGAC)

Dunnett t (2-sided)[a]

Dependent Variable	(I) Conc	(J) Conc	Mean Difference (I-J)	Std. Error	Sig.	95% Confidence Interval Lower Bound	95% Confidence Interval Upper Bound
U251	.10	.00	-4.33778	3.78728	.626	-15.2848	6.6093
	1.00	.00	-9.99958	3.78728	.075	-20.9466	.9475
	10.00	.00	-12.80319*	3.78728	.022	-23.7503	-1.8561
	100.00	.00	-44.88343*	3.78728	.000	-55.8305	-33.9364
U373	.10	.00	1.81986	3.33573	.948	-7.8220	11.4617
	1.00	.00	-10.34447*	3.33573	.035	-19.9863	-.7026
	10.00	.00	-14.56109*	3.33573	.005	-24.2030	-4.9192
	100.00	.00	-42.93729*	3.33573	.000	-52.5792	-33.2954
U87MG	.10	.00	1.97032	6.46560	.993	-16.7184	20.6590
	1.00	.00	-2.91206	6.46560	.973	-21.6008	15.7766
	10.00	.00	-11.69809	6.46560	.272	-30.3868	6.9906
	100.00	.00	-14.85788	6.46560	.130	-33.5466	3.8308

Multiple Comparisons for Figure 5 (Prolipo)

Dunnett t (2-sided)[a]

Dependent Variable	(I) Conc	(J) Conc	Mean Difference (I-J)	Std. Error	Sig.	95% Confidence Interval Lower Bound	95% Confidence Interval Upper Bound
U251	.10	.00	-.72507	2.55659	.995	-8.1148	6.6647
	1.00	.00	-28.81015*	2.55659	.000	-36.1999	-21.4204
	10.00	.00	-58.58090*	2.55659	.000	-65.9707	-51.1911
	100.00	.00	-95.65454*	2.55659	.000	-103.0443	-88.2648
U373	.10	.00	2.26650	9.84636	.998	-26.1942	30.7272
	1.00	.00	-3.49297	9.84636	.988	-31.9537	24.9677
	10.00	.00	-13.12409	9.84636	.509	-41.5848	15.3366
	100.00	.00	-92.92765*	9.84636	.000	-121.3883	-64.4670
U87MG	.10	.00	16.11272	22.22761	.877	-48.1358	80.3612
	1.00	.00	3.15460	22.22761	1.000	-61.0939	67.4031
	10.00	.00	-.92684	22.22761	1.000	-65.1753	63.3216
	100.00	.00	-88.71596*	22.22761	.008	-152.9644	-24.4675

Multiple Comparisons for Figure 5 (Prolipo + XGAC)

Dunnett t (2-sided)[a]

Dependent Variable	(I) Conc	(J) Conc	Mean Difference (I-J)	Std. Error	Sig.	95% Confidence Interval	
						Lower Bound	Upper Bound
U251	.10	.00	3.93695	4.27133	.767	-8.4093	16.2831
	1.00	.00	-16.05215*	4.27133	.012	-28.3984	-3.7059
	10.00	.00	-38.69936*	4.27133	.000	-51.0456	-26.3532
	100.00	.00	-96.85340*	4.27133	.000	-109.1996	-84.5072
U373	.10	.00	6.35972	6.90717	.768	-13.6053	26.3248
	1.00	.00	4.11658	6.90717	.932	-15.8485	24.0816
	10.00	.00	-14.58504	6.90717	.174	-34.5501	5.3800
	100.00	.00	-95.67989*	6.90717	.000	-115.6449	-75.7148
U87MG	.10	.00	1.63113	7.81793	.998	-20.9664	24.2287
	1.00	.00	.01949	7.81793	1.000	-22.5781	22.6171
	10.00	.00	-6.74782	7.81793	.802	-29.3454	15.8498
	100.00	.00	-86.11320*	7.81793	.000	-108.7108	-63.5156
